# Experiences of barriers and facilitators in mental health care transitions: A qualitative exploration of perspectives from transitional-aged youth, family, and service providers (part 1)

**DOI:** 10.1016/j.hctj.2024.100087

**Published:** 2024-11-27

**Authors:** Roula Markoulakis, Hinaya Cader, Karen Wong, Sugy Kodeeswaran, Tracey Addison, Cathy Walsh, Jocelyn Charles, Amy Cheung, Deepy Sur, David Willis, Anthony Levitt

**Affiliations:** a Sunnybrook Research Institute, Canada; b University of Toronto, Canada; c Sunnybrook Health Sciences Centre, Canada; d Family Advisory Council, Family Navigation Project, Sunnybrook Health Sciences Centre, Canada; e Ontario Association of Social Work, Canada; f Strides Toronto, Canada

**Keywords:** Transitional-aged youth, Caregivers, Families, Service providers, Mental health, Addictions, Transitions in care, Continuity of care, Mental health services

## Abstract

**Introduction:**

Transitional-aged youth (TAY) are at a vulnerable stage of their development in which mental health and/or addiction (MHA) issues tend to manifest and/or increase in severity. TAY also tend to find themselves subject to multiple care transitions, often resulting in sub-optimal access to MHA services. The objective of this study was to explore the perspectives of TAY, family members, and system providers regarding the supports needed by TAY and their families during transitions through MHA care.

**Methods:**

This is a descriptive qualitative study of TAY, family, and provider perspectives on their experiences with accessing/providing MHA care and transition supports for TAY. Focus groups and interviews were conducted with 14 TAY, 26 family members, and 23 service providers. Participants were asked about their experiences with regard to barriers and facilitators to transitions in care for TAY with MHA concerns. Data was analyzed utilizing a thematic analysis approach.

**Results:**

Six themes emerged during data analysis: pathways to care, appropriate and comprehensive care, continuity of care, informed care, family involvement, and TAY involvement. These results provide a better understanding of the needs of TAY and their families in relation to accessing and transitioning through MHA system supports and improving MHA outcomes. They also include the views of service providers on the current state of access to and transitions through MHA care, specifically for the TAY population.

**Discussion:**

This information reveals the supports needed by TAY and their families along with the challenges created due to a lack of guidance, transition preparation, collaboration, and continuity in the MHA system. MHA providers working with TAY and families can utilize these findings to promote effective TAY and family engagement for positive transitions and care experiences.

## 1 Introduction

Transitional-aged youth (TAY), typically between 12 and 29 years of age,^1^, ^2^ experience many changes in social, developmental, biological, and psychological domains.^2^, ^3^ They are also particularly susceptible to the first emergence of mental health and/or addictions (MHA) issues or an increase in severity or complexity of these concerns.^2^, ^3^, ^4^ These difficulties often persist, in that 70 % of adults trace their MHA concerns back to adolescence.^5^ While MHA concerns are estimated to impact 1 in 5 youth, fewer than 20 % of youth affected receive appropriate treatment.^6^

TAY with MHA issues face numerous social, emotional, financial, and systemic barriers to care, as well as multifaceted needs that affect their transitions through the many elements of MHA care systems.^7^ The transitions they face may be universal, such as developmentally-related transitions, or driven by local contexts, such as features of local healthcare system policies and practices. TAY are vulnerable to care interruptions, as they may move between many siloed levels and types of care and systems, including child and adult systems or public and private systems, acute care, emergency departments, hospital, outpatient, primary care, live-in treatment, and mobile crisis response.^8^ They may encounter difficulties connecting with appropriate expertise or intensity of support, limiting their participation in effective treatment.^9^ ^10^ Furthermore, while the importance of preparedness for transitions in care is often recognized, transition planning is often lacking or not conducted at all.^11^ Negative experiences of transitions can unfortunately result in complete disengagement from MHA care.^3^, ^12^ Families can be active in driving the care plan and facilitating access to care, encouraging help-seeking, and initiating referral to MHA services.^13^, ^14^ Family participation is variable. Some families may be heavily involved in TAY care, others may promote TAY independence while remaining available to support, while others may be entirely unable to support the youth due to confidentiality or their own needs, circumstances, and backgrounds.^12^

Given the complex and varied nature of transitions that TAY and families may encounter, it is important to understand the perspectives of TAY, families, and service providers regarding the barriers that exist to successful transitions and related access to care, as well as the ways in which these experiences can be improved. Corroboration of these accounts can serve as a frame of reference when developing services and supports that target transitions in care for TAY and families. Thus, the aim of his study was to explore the barriers and facilitators experienced by TAY with MHA concerns, family members, and service providers as they pertain to transitions in MHA care. Part two of this study specifically explored the role of navigation to address the needs identified in the current paper.

## 2 Methods

The current study utilized a qualitative descriptive approach to explore TAY, family, and service provider perspectives of what is needed to effectively access and transition through MHA care.^15^ The research question guiding this study was: what are the perspectives of TAY, family members, and service providers regarding the supports needed by TAY and their families when transitioning through MHA care? All aspects of the study were approved by the Sunnybrook Health Sciences Centre Research Ethics Board.

### 2.1 Setting and participants

Participants were current and former clients and service providers of a youth and family navigation service based in an academic health sciences centre in Toronto, Ontario (i.e., the Family Navigation Project)^16^ and other community services both with and without navigation supports (e.g., East Metro Youth Services, Canadian Mental Health Association branches across the province). TAY aged 13–26 years old and living in Ontario who have experienced or are currently experiencing MHA concerns were recruited (with no requirement of formal diagnosis); family and service providers for such TAY were also recruited. TAY-family dyads were not specifically sought or required for participation. Potential participants were invited to participate in this study through the distribution of recruitment posters. Interested individuals reviewed an information letter outlining the details of the study and provide informed consent to participate. Participants were asked to indicate preference to participate in an individual interview or focus group and whether they preferred to participate in person or via telephone. The study interviewer and participant then scheduled a time and date based on the participant’s preference.

### 2.2 Data collection

Data was collected through semi-structured individual interviews and focus groups from January to May 2020 via telephone or in person. All focus groups and interviews were conducted by the same interviewer – the study research assistant (HC). Focus groups were co-facilitated on a rotating basis by an additional member of the research team (n=3), who represented the participant group (i.e., service provider, caregivers). Participants were first asked to complete a demographics questionnaire inquiring about age, gender, location, participant type (i.e. TAY, family, or service provider), and current level of education. In the questionnaire, TAY participants were also asked about their current living arrangements and their opinion on level of family involvement in their care. Service provider participants were asked to provide the number of years they have worked with TAY with MHA concerns. During interviews, participants were asked to describe experiences when trying to find MHA services, what TAY and family needs are regarding transitioning through MHA supports, etc. The interview guide was developed based on initial literature review as well as group discussion and revision with the full author team. Questions were tailored to the specific respondent group (i.e., experiences seeking services for their youth, when speaking with family; experiences delivering services, when speaking with service providers). See Supplement 1 for a sample semi-structured interview guide, tailored for one-on-one interviews with TAY. All interviews and focus groups were audio recorded and transcribed. Throughout the data collection period, the study interviewer met often with the study lead (RM) to reflect on emerging data and revisit the interview guide as needed.

Data was collected with 63 participants in total: 14 TAY, 26 family, and 23 service providers. Three in-person focus groups were conducted: one with FNP staff participants (n=4) and two with family caregiver participants (n=3 each). These ranged in length from 109 to 115 minutes. 53 individual interviews were conducted: 3 in person and the remainder via telephone, largely due to the onset of the COVID-19 pandemic (n=50 by telephone, 12 of which took place prior to the pandemic). The interviews ranged in length from 23 to 96 minutes.

### 2.3 Data analysis

A thematic analysis approach was used to analyze data obtained from the focus group and interview transcripts.^17^ All transcripts were first read through by coders to gain familiarity with the content. Three members of the research team (HC, RW, and RM) reviewed nine transcripts (three from each participant group) to develop initial codes from the data and then met to discuss code definitions and to generate a preliminary codebook which would guide the coding of remaining transcripts. One codebook was used for all participant groups. All coding was conducted using MAXQDA 2020. As coding proceeded, this codebook was then revised as needed and the coders met regularly to discuss codes and track changes to the codebook, thereby maintaining an audit trail and allowing previously coded interviews to be updated in line with revised codes. Analytic memos were used throughout the analysis process to note their interpretations that arose while coding, and were then discussed as a group during ad hoc analysis meetings to share perspective on the data and triangulate findings.^18^ Emerging findings were also discussed as a group and codes were sorted into potential themes and sub-themes. The coded transcripts were then reviewed to ensure the developed themes accurately reflected the data and were applicable to all respondent groups, after which the themes were refined, named, and analytically defined. Finally, findings from this analysis were written into this report.

## 3 Results

See Table 1 for information about participant background. Participant groupings are indicated next to the participant number (TAY, family (F), or service provider (SP)) following sample quotes. Six themes emerged during the data analysis process: *pathways to care*, *appropriate and comprehensive care*, *continuity of care*, *informed care*, *TAY involvement*, and *family involvement*. See Table 2 for a description of each of these themes and respective sub-themes. Below, descriptions of each theme and subtheme along with participant quotes are presented. Although participants described barriers and facilitators in their experiences, the themes and subthemes are presented in terms of the resultant needs that exist for TAY and families as they transition in MHA care. Depending on how the study themes and subthemes were enacted, they could present as barriers or facilitators in participants’ experiences. Thus, whether and how these needs are addressed can create barriers or facilitators for TAY and families as they encounter MHA care transitions.

**Table 1:** Description of Participants.

**Participant Type**	**TAY (n=14)**	**Family (n=26)**	**Service Provider (n=23)**
Average age (yrs)	23	54	36
Women %	71	88	91
Location	Toronto = 10 Greater Toronto Area (GTA) = 4	Toronto = 14 GTA = 8 Other – Ontario = 2 Unanswered = 2	Toronto = 17 GTA = 3 Other – Ontario = 2 Unanswered = 1
Highest completed education	Secondary = 2 Postsecondary = 11 Postgraduate = 1	Postsecondary = 18 Postgraduate = 6 Unanswered = 2	Postsecondary = 4 Postgraduate = 18 Unanswered = 1
Relationship to TAY		Mother = 22 Father = 3 Grandmother = 1	
Average # of years worked with TAY			10 years
% caseload working with TAY			<75 % = 1 >75 % = 22
Living arrangement	At home with family = 11 Independent = 3		
Family involvement in care	Not at all involved = 4 Slightly involved = 6 Moderately involved = 3 Very involved = 1

**Table 2:** Themes and sub-themes regarding transitions in care for TAY with MHA concerns and their families.

**Theme**	**Description**
Pathways to care (3.1.)	TAY and families encounter multiple entry and exit points in their care, barriers to services, and unclear pathways as they transition through care. TAY and families may not be aware of entry points into MHA care, paths between one service and the next, and means of accessing services. These experiences affected and were affected by the *timeliness of help-seeking (3.1.1.)*, the *accessibility of care (3.1.2.)*, and subsequent strain and frustration due to the lack of *clarity of care pathways (3.1.3.)*.
Appropriate and comprehensive care (3.2.)	TAY and families experience difficulties identifying and connecting with care that is appropriately tailored to their individual concerns and comprehensively addresses the full range and extent of their needs. TAY and families encounter supports in the MHA system that do not always provide *appropriate care (3.2.1.)* to address their needs. They perceive a need for *flexible care (3.2.2.)* and *comprehensive support (3.2.3)* offered by *providers with skills and approaches (3.2.4.)* that demonstrate an appreciation for the particular needs and preferences of TAY.
Continuity of care (3.3.)	TAY and families encounter numerous transitions in MHA care, during which continuity of care is often disrupted. TAY and families have a need for *uninterrupted care (3.3.1.)*, particularly in times of transition. The availability of *continuous supports (3.3.2.)* and an individualized *focus on transition preparation and readiness (3.3.3.)* is important to ensure seamless transitions.
Informed care (3.4.)	TAY and families experience inadequate availability of information to make informed choices about their care, or are uncertain about where to seek and find appropriate and reliable information to support their care decisions, particularly when transitioning between services. They are desirous of *access to resource information (3.4.1.)* about care options and meaningful relationships with providers for *guidance (3.4.2.)* regarding their care options. These supports can enable them to have informed *choice (3.4.3.)* in their care.
Family involvement (3.5.)	Families may become involved in TAY MHA care and can provide a range of supports to facilitate TAY access to and transitions through MHA care. Namely, they can provide *practical support (3.5.1.)* and *soft supports (3.5.2.)* for TAY. However, families may also experience *caregiver strain (3.5.3.)* while having to manage multiple aspects of TAY’s care and may face *confidentiality (3.5.4.)* issues as TAY transition to adulthood.
TAY involvement (3.6.)	Actively involving TAY in their MHA care is important throughout the care trajectory and especially during times of transition. There are numerous factors that can affect TAY *participation in care (3.6.1.).* TAY MHA care should consider the youth's preferences and needs, with providers and services operating in a manner that provides *TAY-friendly care (3.6.2.)* to encourage their participation. Ultimately, these efforts can result in growing *independence (3.6.3.)* in their care, especially when family cannot be involved in care.

### 3.1 Pathways to care

#### 3.1.1 Timeliness of help-seeking

Participants noted a delay in initiating care pathways for MHA concerns. One TAY described how these concerns can be missed, stating that *“it’s easy to mask when parents are uneducated about these sort of things…they [don’t] recognize it as a problem”* (066-TAY). A caregiver noted that parents may disregard MHA symptoms as common adolescent behaviours: “*we were pulled aside by one school administrator who was concerned about his drinking…we kind of brushed it off at that time, thinking it’s just a kid who is rebelling and it’s a phase that many kids go through*” (005-F). Even after detecting the presence of MHA symptoms, some TAY may be reluctant to access support, possibly arising from concern about “*the optics of receiving therapeutic supports”* (048-SP), fear or mistrust of MHA care, or even the stigma associated with MHA. One TAY shared how their family discouraged them from speaking up about their MHA concerns: *“she has said ‘if you have a problem, don’t tell anyone about it and act like you don’t have one’”* (006-TAY).

#### 3.1.2 Accessibility of care

TAY and their families may have difficulties accessing care when faced with transitions, due to a number of factors affecting availability and equitable access in the MHA care system. The lack of resources, specifically due to limited staffing or service capacities, was one perceived cause. One TAY participant stated:

*“If my adult psychiatrist had a social worker or had a therapist on his team…that would’ve been a place for me to go. But he was on his own… he is only one person and I am sure he had hundreds of people who want to talk to him”* (050-TAY).

Consequently, many participants described extensive wait times for care. A service provider described how wait times can be problematic and create multiple transitions: “*because of such a long waitlist, we often see our youth relapsing or losing contact with us. And then we have to start all over*” (001-SP).

Participants also expressed the need for more convenient geographical location of services and less restrictive catchments. One participant suggested that traveling far for support might compound youth’s MHA issues: “*we should have access to things that don’t require an hour drive downtown for a kid with anxiety who already can barely leave her neighbourhood*” (063-F).

Inequities in care were also identified, highlighting the need for culturally responsive supports that can facilitate greater access to MHA care for a more diverse group of people. One participant stated, “*I still, every day, see racism when I see youth of visible minority trying to access medical care, especially for mental health or addictions*” (001-SP). Another participant voiced a similar concern, noting that “*a lot of communities may not have the same access, like racialized communities, [newcomer] communities, LGBTQ2+ communities, Indigenous communities*” (039-SP). However, accessing culturally responsive supports was important to help address individual needs: “*I wanted not only to talk about some issues relevant to my cultural background but actually go with my mom for a few sessions. And so it was a matter of finding someone who could not just understand that perspective but actually spoke our language*” (022-TAY).

Restrictive eligibility criteria were also identified as a common contributor to inaccessibility. Participants shared that rigid age thresholds often resulted in being removed from care, especially when TAY turn 18, “*they say, ‘oh sorry, she’s going to turn 18 soon, and then we can’t help you.’…But then you can’t get on the list for the over 18 services because they’re under 18. So you have to wait*” (019-F). Participants also shared instances of TAY aging out while on extensive wait lists and then needing to begin again on a new waitlist for an adult service, which further impeded their access to care.

These limitations also present in terms of eligibility for service. As one caregiver was told, “*‘we can’t help you because your daughter hasn’t hurt anyone, broken the law, or tried to kill herself’*” (019-F). Another frustrating aspect of eligibility that participants expressed was the need for formal referrals and diagnoses to be able to access care. One TAY commented on the difficulty that these requirements can cause, especially when trying to access publicly funded services:

*“a lot of publicly funded services need doctor’s referral, but that is not the easiest thing to get. And then there is also the issue of some publicly funded services that require you to have a formal diagnosis of a mental health illness, but getting a diagnosis can take a long time and be difficult”* (037-TAY).

Participants indicated the financial cost of services prohibited access to MHA care, demonstrating the need for more affordable and/or free options. This was especially the case when TAY tried to access private supports in efforts to avoid the wait lists associated with publicly-funded supports, as one service provider stated: “*if you are waiting for a publicly funded bed…you are going to be waiting 18 months. Versus if you are going to be able to do a fee-for-service bed…you are going to be able to get there within 2–3 months*” (046-SP). Insufficient insurance coverage or limited financial resources were common reasons for making a conscious decision to access mental health supports less frequently than necessary, as one TAY shared “*thankfully I can pay for it but I can’t do it weekly which I would like to*” (034-TAY).

#### 3.1.3 Clarity of care pathways

Participants shared that pathways to care are unclear, often making it difficult to find one’s way through the MHA system. As one participant lamented, “*where do you go to decide where to go?*” (004-F). A caregiver noted the paucity of information regarding available care, stating, “*I don’t know what’s out there…it’s not a straightforward map, it’s not a chart that’s already lined up for me*” (057-F). In fact, some participants indicated that they felt they were able to find the right supports by chance alone, stating, “*I feel like you just almost accidentally stumble upon something that’s useful*” (062-F), or “*that was kind of a fluke*” (059-F), and “*it’s literally based on my pure luck*” (040-SP).

The difficulties associated with navigating the MHA system leave people feeling hopeless and frustrated. One caregiver stated:

*“I meant to use the word frustrating but it doesn’t really capture the despair of watching your child struggle with a life-threatening illness and there is no one to turn to and say, what can we do to help this child recover”* (023-F).

Another caregiver associated the hopelessness and frustration experienced when trying to navigate the system with his daughter’s death:

*“My own challenges were just sitting in waiting rooms with [TAY] hoping that this was going to be the one that cracked it, just for someone to come out and be like, ‘oh sorry, we’re not going to be able to help’ for whatever the litany of reasons was. We got a long way down a lot of garden paths just to find a closed door at the end. To say that was frustrating would be a deep understatement and maybe, most damagingly, it was constantly sucking hope out of [us] parents, which is the worst part. It’s why she’s dead. Couldn’t give her enough hope. And she just didn’t see it getting better”* (055-F).

In response to the unclear care pathways for TAY with MHA concerns described by participants, they also highlighted a need for coordination of care. Speaking from the service provider perspective, one participant described the lack of collaboration as “*all kind of shooting in different directions and not working together*” (036-SP). Similarly, one participant noted that service providers also feel lost within the system:

*“It’s surprising how much we don’t know about our neighbours… we don’t know about the other services that exist around us. So even when we are well-intentioned to help…we sometimes are also feeling in the dark, trying to understand the pathway”* (041-SP).

One participant stated, “*it just seems like every service provider is independent of one another whereas I think it would be more effective if they could all talk to one another and have access to information*” (037-TAY).

### 3.2 Appropriate and comprehensive care

#### 3.2.1 Appropriateness of care

Participants shared the care TAY receive is often not tailored to their individual needs. They may receive care that is too intense or specialized or even not specialized enough. TAY needs may be particularly complex due to care and life transitions, such that “*their life needs are so complex in themselves that sometimes they don’t fit into the box of what’s being offered*” (047-SP).

Participants indicated that some supports focus on symptom resolution rather than addressing underlying MHA concerns. Participants especially viewed this to be the case in crisis situations where TAY are hospitalized and stabilized before being discharged without any further support. One caregiver participant stated, “*when there is a crisis situation, there is no support there. They treat the crisis, 24 hours later, they say okay, everything is fine, he has calmed down, he can go home and that’s the end of the case*” (005-F). Similarly, one participant described the inevitable recurrence of crisis when underlying needs are not met:

*“the crisis was dealt with, he was released back into the wild, into the exact same environment, school-wise, socially, domestically at home, as pre-existed. So with no changes, we can come to a conclusion that this would simply just happen again. And it did”* (002-F).

This often leaves TAY with no choice but to access a support that they know may not necessarily be helpful. One participant expressed, “*because I didn’t see a service for me out there, I just took whatever services I could get…which was not treating the actual problem that I had*” (050-TAY). These experiences resulted in TAY attempting to access multiple supports to increase their chances of receiving appropriate care, which was likened to being “*stuck in a vicious cycle of couch-surfing through medical providers*” (001-SP), thereby precipitating a negative cycle of repeat care transitions.

Participants also shared instances where TAY felt out of place in supports available through both the child and adult care systems, highlighting the need for age-appropriate supports: “*I’m an adult, but I’m being treated like a child because that’s the only group that they know how to work with*” (006-TAY). Similarly, another TAY participant shared, “*I was with a lot of kids…there is a lot of authority and babysitting kind of vibes*” (024-TAY). Participants expressed the same concerns regarding TAY participating in supports aimed at more mature adults. One caregiver shared,

*“if he is going to be in the room with adults who are 45 years old, and they are talking about divorce and alcoholism, all the other things that they are bringing to the table as an adult, it’s not the same experience as an 18-year old”* (032-F).

When care was not appropriate or structured in a manner that was friendly and supportive to TAY, they felt disengaged. One TAY described such an experience: “*I just felt like it was more of a chore or homework…It didn’t really feel like that was doing much for me*” (066-TAY).

#### 3.2.2 Flexibility in care

TAY with MHA concerns need flexibility in both the way they receive care as well as when supports are available to them. While some participants preferred in-person care options, participants also appreciated technological solutions that make care more flexible and accessible, noting, for example, that TAY “*would rather text or email*” (033-SP). Another participant shared that having a virtual option made it possible to balance their care with a lengthy commute:

*“I get home pretty late in the evening so if I were to add on going somewhere else to see a counsellor during the week, I think that would probably prevent me from reaching out as much and I probably wouldn’t go as often”* (022-TAY).

Limited service hours were also raised as a target for greater flexibility to enable TAY to access supports. Services that are offered only during weekdays often interfere with TAY school hours, leaving them stuck between choosing to prioritize either school or their MHA concerns, noting that many TAY “*don’t want to leave school to make these appointments. So then they struggle through with their mental health*” (001-SP). A TAY participant also described this concern:

*“I wish some services were open later just because I’m in school during the day…if I want to go see this person, I have to go in between classes and risk being late to my second class. Or totally miss a class and book the appointment for that class time, and just skip a class, which is never a good thing to do”* (006-TAY).

Caregivers were also affected by limited service hours, due to the role they can play as a means of transportation for TAY, with one participant stating: “*there are not a lot of after-hours services where parents aren’t having to lose pay in order to take their kids to appointments*” (012-SP).

#### 3.2.3 Comprehensiveness of support

TAY with MHA concerns require a comprehensive and holistic approach to address the many aspects of their lives where they may be experiencing transitions, so that TAY and families are “*not running in a hundred directions trying to pull all the pieces together*” (032-F). For example, participants were desirous of supports that can help TAY achieve independence with regard to academics and their careers, ideally “*someone [who] can guide them to do the resume, find the passion, and how to guide them to get the education, and lead them into maybe a part-time job, to be independent*” (018-F).

Psychoeducation to enhance MHA knowledge was also identified as a much-needed component of comprehensive care that could improve access to MHA supports. Participants suggested that providing psychoeducation in places where TAY already are, such as schools, could facilitate early intervention for MHA concerns. However, they also noted this was not yet the case, stating “*I don’t think that having a mental health conversation at school was a universal experience. It was that one teacher that was really passionate about it…but it wasn’t something that the school would prioritize*” (022-TAY).

Participants also recognized that families of TAY with MHA concerns can especially benefit from psychoeducation in order to better support their TAY through accessing and transitioning through care. One participant stated, “*families don’t necessarily know why their youth or child is acting out…and [see] it as behavioural issues rather than a mental health issue*” (012-SP). Families could benefit not only from psychoeducation, but also additional family supports “*to have someone listen to them, to have someone give them perspective, that what they’re doing is supporting their youth, and then also how can you build on what they’ve already been doing*” (027-SP).

For TAY, support from peers was valued as an asset to accessing MHA supports, specifically support from peers with experience in transitions in MHA care. One participant suggested a “*peer support network for young adults, so there is a group where there are no professionals so it feels like there is a discussion among peers*” (024-TAY). Participants also suggested that TAY are receptive to support and recommendations from youth peers: “*using things like peer mentors or using youth advocates to say, ‘hey, I used this service and this worked really, really well for me.’ I think that speaks volumes.*” (001-SP).

#### 3.2.4 Skill and approach of service provider

Since service providers play a crucial role in supporting TAY with MHA concerns, their skills and approaches to care were identified as an important mediator for transitions in care. One participant explained, “*they were awful. I mean, really not sympathetic. Not understanding, not willing to help us find the alternatives because she was going to age out*” (019-F). These difficulties were also encountered as a result of what was perceived as role constraints and/or inadequate MHA system knowledge. One participant stated, “*I think sometimes family doctors are willing to do it. They just don’t know what they’re supposed to do and how they’re supposed to do it. But yet they hold all of the information*” (027-SP). Another participant stated their family doctor “*didn’t know anything about the mental health system*” (011-F).

Participants also voiced concerns regarding service providers’ approaches with TAY, indicating a desire for more effective and youth-friendly communication. One participant described how a service provider threatened a TAY: “*rather than meeting this youth where they are at, she has to exert her authority and say that she can sedate them basically if they didn’t calm down*” (036-SP). A caregiver described an incident with inappropriate communication and lack of compassion following an instance of self-harm:

*“he said, ‘I don’t think she was very serious about it, they’d be much deeper cuts if she meant it.’ And I just grabbed him and pulled him around the curtain and said, ‘hey you can’t be this stupid.’ She was in there screaming, ‘you want me to [expletive] try harder?’”* (055-F).

In contrast, participants shared occurrences of compassion and understanding from providers, and how important this was to bridge interruptions and transitions in care:

*“in between periods when he didn’t have a psychiatrist, his family doctor was prescribing his medication and acting almost as a bit of a therapist for him…But it wasn’t at the level that you’d need for someone like [my son]. But he was a compassionate ear for my son and he would try and encourage him to stay on his medication and get help and do what he needs to do”* (058-F).

Some TAY and caregiver participants voiced their appreciation of service providers who are sympathetic to TAY experiencing transitions in care, as one noted:

*“[they were] very conscious of the fact that sometimes people have used services before and that having to repeat yourself and go over things again can be a difficult thing…I found them to be helpful”* (037-TAY).

### 3.3 Continuity of care

#### 3.3.1 Uninterrupted care

TAY experience a number of interruptions in their MHA care pathways, especially at the transition boundary. Participants noted this issue with regard to waitlists and age-related transitions:

*“Because of long waitlists, particularly in public services, let’s say a youth is transitioning into adult services at age 18…[if] they didn’t know they need to get on a waitlist at 16, they may have a gap with a few years of not even having any service”* (026-SP).

These care interruptions can occur quite abruptly, without preparation or awareness:

*“then she turned 18, it was like they all disappeared in the blink of an eye. And then that’s when she got much worse, after 18. So it was at the time when we needed the scaffolding, the pathway, the compassion, all of those pieces…were suddenly, it was kind of on your own to figure out” (013-F).*

Approaching such transitions can feel like “*starting from scratch*” (022-TAY). For example, one service provider shared their perspective on caregivers’ feelings that can accompany this important transition:

*“to think, ‘oh my gosh, I have to do this all over again.’ Knowing that their youth is still going to present in the way they presented last year even though they’re a different age. And they have to now learn this whole new entire system on their own. That can be very overwhelming”* (027-SP).

#### 3.3.2 Continuity of support

In light of the identified need for uninterrupted care, participants expressed the importance of the availability of longer-term and more continuous supports. Participants described experiences with short-term services and/or appointments of insufficient length or frequency, noting, “*you find somebody and then you get 12 sessions because everything is short-term and then you’re done. And [then] you have got to find someone new*” (018-F). Another caregiver agreed, “*we thought we could finally get some help, but that doesn’t last*” (021-F). Repeat transitions also further diminish what are already short-term services. One participant shared, “*my first or second sessions, I am normally getting them caught up with where I am at*” (022-TAY). TAY and families experience a great deal of redundancy, constantly retelling their stories at every intake appointment in order to access a new support, finding themselves saying “*the same thing over and over again about a hundred times*” (019-F). Another participant pointed out that this can be “*traumatic having to retell your story a thousand times…traumatizing to relive it over again*” (012-SP).

The lack of assistance with ensuring continuity of care when experiencing transitions often result in TAY and families chaining together services for themselves in an attempt to attain some semblance of continuity. This can be a very taxing activity, which one participant described as “*McGyver-ing it. I feel like I’m a detective. It’s looking for clues here and there, talking to as many people as I possibly can*” (019-F). Such accounts speak to the need for longer-term, more continuous supports. As one participant noted, “*I think a lot of the times people struggle with being able to get consistent care and seeing the same person*” (033-SP). This may also take the form of a support person that can be a source of continuity throughout the transition period between other services. One participant suggested,

*“it’s important to have somebody to be involved to bridge the gap and make sure that whatever services are being provided, the [TAY] is able to access, they make sense, and there is somebody to follow up to make sure they are working out”* (045-SP).

#### 3.3.3 Focus on transition preparation and readiness

TAY require support in ensuring that transitions proceed smoothly, through transition preparation as well as consideration of their readiness for transition. Many participants pointed out, however, that adequate planning for transitions is often not offered, leaving them with nowhere to turn once the services they are receiving come to an end. One caregiver mentioned that when faced with a service transition, their youth “*didn’t leave with a treatment plan or anything, or a referral, nothing…everyone shrugs their shoulders and says it’s a terrible problem*” (017-F). Another participant suggested that service providers may not have the resources to conduct individualized transition planning:

*“They are turning 18 and they are aging out…I don’t know if the staff who do the front line work have enough time to dedicate towards calling and making sure the service is a right fit. So sometimes people are given a list or more general options to access”* (045-SP).

Participants emphasized the important role of early planning to support the transition process. One TAY suggested,

*“starting to say, ‘hey you are [nearly] 18…here’s how we can start preparing you for adult services.’ That would definitely have been appreciated”* (050-TAY).

Preparing TAY by setting expectations about the shift from child to adult services was also highlighted. One participant noted the importance of sharing with TAY “*what they are entitled to, what they are no longer entitled to, what they gain by being an adult*” (050-TAY). Participants viewed this as relevant in terms of the possible inability of adult services to include family in the TAY’s care. As one participant noted: “*adult services aren’t set up for a parent to be doing the planning and organizing and that can be quite a change*” (064-SP). Participants also viewed advance transition planning as critical to preparation, so that “*you are not just left to your own devices, not knowing where to go next*” (014-F). One participant highlighted warm transfers as one way of accomplishing this:

*“Making the connections between the current care provider and the upcoming one, making those connections before the actual kind of handover helps everyone get on the same page and helps the youth. The young person gets comfortable with the new person versus it being a sharp change”* (064-SP).

In order for TAY to be effectively prepared for transitions in care, their readiness for transition must be considered and supported. One participant pointed out the importance of readying TAY for transitions: “*oftentimes when they’re not ready, then they’ll just leave. So if you have that prep time with them, they know what to [expect]*” (001-SP). Furthermore, recognizing that TAY are often at a point in life where multiple transitions may be occurring simultaneously, it can be important to assess transition readiness to minimize disruption in what can be a particularly stressful period in their lives. One participant further described how a shift into adult services can be unsettling for TAY:

*“They may also be away from family now for the first time in their life and so they don’t have that extra pair of eyes on them if they’re struggling. And even though they might be treated as an adult within the systems, they might feel they could benefit from having people that know them well around them”* (026-SP).

Finally, participants noted that transitions based solely on age might not be effective as a whole. Systemically mandated age-related transitions do not account for the developmental readiness and ongoing needs of TAY, as “*it’s just the birthday that went by, the child hasn’t changed that much, 17 to 18, in terms of their needs*” (064-SP).

### 3.4 Informed care

#### 3.4.1 Access to resource information

Participants indicated a need for resource information to be easily available to TAY and families to ensure seamless access to care at the interface of care transitions, given that “*you don’t know what you don’t know*” (014-F). When attempting to investigate what services might be available for their needs, participants shared that the right information might not find its way to the right person. One TAY participant stated, “*we never got any information that such a thing even existed*” (068-TAY). Available service information was also described as difficult to filter through, as one participant noted, *it’s just so scattered. And trying to find things, it’s really difficult. And when you find them, weeding out the vast majority*” (054-F). TAY and families take it upon themselves to piece together information from various people and providers in the MHA system, in hopes of coming across something worthwhile. One participant noted: “*you talk to your friends, you talk to family members, I know this person, they were here, they did that, that seems to be the way information has dropped into my lap*” (032-F). This approach was also seen to be highly inconsistent, depending on *“who happens to know about a certain resource. It’s very hit and miss*” (054-F).

The general perception of an insufficiency of resources across the MHA system was thought to be driven in part by the difficulties experienced in locating appropriate services. Participants stated, “*I found there isn’t a shortage of resources; there is a shortage of knowing how the resources differ from each other*” (040-SP) and “*there’s a ton of things available that not everybody knows about.*” (065-SP). This speaks to a need for increased awareness and understanding of available supports, as one participant highlighted: “*it would be a lot easier to get people to access those services…if there [was] information more readily availa*ble” (006-TAY). To increase awareness of supports, information should be widely distributed, especially in places frequented by TAY; for example, one TAY suggested, “*getting it into schools, sending it out in newsletters, going into community centres*” (022-TAY).

Due to the lack of accessible information and lack of awareness of the availability of such information, TAY and families often resort to searching desperately online for service options. One navigator described this issue, stating, “*families don’t know where to go so they have to do their own research to figure out where to go for mental health services and they are [searching online] and making phone calls*” (045-SP). However, even independently searching for information could present limitations, as one caregiver explained:

*“you can be so stuck forever because you don’t have basic information. And even if you seek out basic information online, there’s a lot of other knowledge that is not shared in that way, that is known by the people who understand the system, who have connections, who have the ability to put in the word, who have the ability to recommend you to stuff”* (013-F).

Participants strongly felt that not only should TAY and families have better access to knowledge about the MHA system, service providers need to remain updated on the availability of resources in the system. Family doctors in particular were identified as providers that could play a key role in providing TAY and families with resources, with one participant stating “*it would be good for family doctors to have more resources or to be more aware of the resources available in the community*” (008-F). However, this is often difficult, as medical practitioners, service providers, and other professionals may be unaware of supports. These sentiments identified a need for specialized services to help find information about MHA supports, as “*it would be very helpful if we had more support services that can help us find possible treatment, people, programs that are out there*” (063-F). However, participants stressed that “*you cannot replace the human touch*” (008-F) in such approaches.

#### 3.4.2 Guidance

Not only do TAY and families need access to information about supports and the opportunity to make their own choices regarding supports, they also require guidance on how to use this information and make the appropriate choices for TAY MHA care. Several participants voiced their frustration with the lack of guidance available to navigate MHA supports and transitions through the system. For example, one participant stated, “*I just didn’t know what to do and could not find anybody who would sit down with me and say, this is the kind of care your daughter needs and this is how we are going to handle it*” (023-F). TAY and families might not have the system knowledge needed to understand and interpret their different care options, as one participant explained, “*what is a social worker, what is a psychologist, what is a psychotherapist, what’s a counsellor, what’s an addiction worker, so on and so forth. What does each person do and what’s within their scope*” (046-SP). A TAY similarly stated: “*I don’t even know where to start or what I need. Do I need to see a psychologist, do I need to see a psychiatrist, do I just need to go to a social worker?*” (066-TAY). Thus, guidance is especially critical to help TAY identify resources to best suit their needs. Another TAY voiced a desire for guidance: “*someone…to be able to [point] you in the right direction and say here are the options, I think would be incredible*” (022-TAY). A caregiver agreed, “*I didn’t really have anybody who was able to sort of sit down and sort of say, ‘okay go here, go there.’ I think we need more of that*” (058-F).

Not only do TAY and families need guidance, they need to have a caring touchpoint to provide this guidance for them. Participants expressed a preference for guidance in the form of live, human support rather than impersonal online guidance. One participant explained,

*“Having someone help guide you through and even holding your hand to show you what can be done to get you help or get you into group or activities where you can talk and get the help you need. It’s just extremely important that they are there to help you through that time”* (049-TAY).

These caring touchpoints can provide the navigation support that participants highlighted is much needed in the MHA system, considering the fragmentation of supports identified earlier. This is captured in one participant’s statement:

*“I think if we had someone to provide navigation support, I feel like I would more likely access help, that it wouldn’t be on me to figure everything out. There will be a professional who clearly understands where to turn to for specific needs”* (025-TAY).

#### 3.4.3 Choice

Participants expressed the need for TAY and families to have a choice in their care when accessing and transitioning through the MHA system. Participants expressed the importance that TAY “*can have some say in where they are going and what they are doing*” (064-SP), so that they are able to choose the care and transition pathways they want to pursue instead winding up in care that would not be responsive to their needs. One participant noted, “*if you’re waiting for a year for public services, whoever has availability, you’re just going to be matched with, you don’t have any choice in that, and it may or may not be a great therapeutic match...*” (026-SP). Participants highlighted the role of providers in ensuring that the information and guidance they provide results in TAY and families exercising choice in their care. One caregiver described their desired experience:

*“you go and ask for help, you are offered and given the information on all the supports that are available. And let it be your decision and if A doesn’t work, then try B, if B doesn’t work, try C. Rather than here’s your counsellor, here’s your date…there you go’”* (061-F).

The ability to make these choices is enhanced when accompanied by guidance (as described in 3.4.2) from service providers, which can allow TAY and families to make the best decisions suited to their needs. One TAY shared their positive experience of being able to collaborate with a provider and family to have choice in their care:

*“he was recommending a bunch of different things. That was pretty useful. Like presenting different options and not just telling me what to do, and allowing my family to be there and have a family consultation kind of thing. I think that’s important for the young adult.”* (024-TAY)

A provider described what this entailed in their service and the importance of this approach for TAY:

*“So youth give us something, a barrier that’s happening in their life, and then we just help give them opportunities and choices about how they can solve it. We never tell them how they need to solve it…Which awesome because then they become their own experts” (001-SP).*

### 3.5 Family involvement

Families can play a considerable role in TAY MHA care. There was a notable perception among participants that family involvement can enhance TAY access and transitions through care, as one participant explained: “*service providers want to have the families involved…because they can make the outcomes a lot better*” (045-SP). However, it is important to note that some TAY may not find it appropriate or productive to have family involved in their care, perhaps due to problematic family dynamics. For example, one participant explained, “*some of the youth we deal with have so much family trauma, or their families are just so unwell that it wouldn’t be helpful to have them*” (028-SP).

#### 3.5.1 Practical support

Families can provide several forms of practical support for TAY accessing MHA care. Perhaps most importantly, they can help TAY find the services they need, as “*parents just have to do a lot of legwork*” (014-F). This can especially be the case when the TAY’s symptoms prevent this involvement, as noted by one TAY participant: “*when I was really unwell, my mom was doing a lot of the research for me*” (024-TAY). Families also may provide financial support for TAY to be able to access care, whether it be through personal savings or parental insurance coverage. A TAY participant shared: “*thankfully myself and my family are in a position that we have some extended health coverage to cover private counselling, but then I was also in a position with my family that we are able to pay for my counselling out of pocket*” (037-TAY). Additionally, families can support TAY by providing transportation to MHA appointments, facilitating access to care that may be geographically inaccessible to TAY otherwise.

Families also check in with service providers when TAY are unable to do so; and they may participate in the TAY’s intake and treatment. One participant stated, “*it’s almost necessary involving family members because they’re the ones that are holding a lot of this health information*” (029-SP). One caregiver participant shared their experience in supporting their child to access care:

*“She would literally sit next to me and she’d be on the phone and she’d be like ‘what am I supposed to say?’…and so I’d have to coach her through all of it because part of the reason we needed these services was because she was so incredibly anxious that she couldn’t do it on her own”* (019-F).

Another caregiver indicated the ways in which caregivers can support TAY by holding and providing information relevant to their care:

*“A lot of the times, he would be in a meeting or something and he just didn’t have the capacity to remember what was being said or so he would rely on me to take notes…Also, there were times where he was in crisis, there was no way that he had the capacity to basically explain his history, medication he has been on, places he has been. So I was lucky that he allows me to be part of it. Because otherwise I don’t know how he would have coped.”* (058-F).

#### 3.5.2 Soft support

In addition to practical supports that facilitate TAY access to MHA care, families can also provide interpersonal supports for TAY. Family involvement in the form of emotional support can facilitate TAY access to and transitions through MHA care, as one caregiver described:

*“It’s just mostly being supportive and listening, and learning that she is still having issues accessing help. And she still is trying to see people, get counselling. Sometimes waiting, or listening to her frustration when she can’t get the help she needs. More being supportive and listening”* (014-F).

Families also play a role in normalizing care, possibly making it easier for TAY to reach out for help when they need it. For example, one TAY pointed out that knowing their father wanted to access MHA care made it easier for them to access care as well: “*he made it clear about, ‘I need to see a therapist too’…I think that does encourage me to continue*” (022-TAY). Similarly, another TAY explained that their family’s normalization of seeking care made them comfortable enough to seek help when needed: “*had I not been in a household where seeking mental health was encouraged…I don’t think I would be nearly as comfortable being able to do it myself*” (050-TAY).

#### 3.5.3 Caregiver strain

Although families are uniquely positioned to provide significant support to TAY, because of the complexities involved with accessing MHA care, families can become overwhelmed with the task. Caregivers shared the sense of powerlessness that stemmed from caring for their youth:

*“I’m not at all an expert and was faced with the most important thing to me in the world, my daughter and her mental health. And despite my education and abilities, I just felt unequipped to know where to go to get her the right help”* (004-F).

Caregivers also shared that despite their efforts, their child’s care was out of their control: “*I felt like many times, my hands were tied. I just couldn’t do a thing*” (005-F). This is especially the case considering caregivers are often balancing this task with other responsibilities at work and/or home. Caregivers noted that they *“had to learn how to navigate the system, which was a full-time job on top of a full-time job*” (013-F). One caregiver participant voiced their frustration with juggling employment in addition to caring for their child: “*it can be very difficult because I am in a professional position…I can’t always in the moment address the support [phoning] me*” (002-F). Such circumstances often result in caregivers prioritizing TAY wellness over their own wellbeing. For example, one participant stated, “*I took time off work, my husband just had to take a leave of absence. It has affected our mental health, it has affected our relationship, it has affected everything*” (010-F).

#### 3.5.4 Confidentiality

As TAY age in an MHA system that tends to form siloes between child and adult services, families encounter several confidentiality issues that may obstruct them from being involved in TAY care. These confidentiality issues often leave families feeling excluded from their TAY’s MHA care. For example, one caregiver participant stated, “*nothing was ever shared with us*” (058-F). Another shared “*I felt like many times my hands were tied. I just couldn’t do a thing, it was all up to [son] because of the age… you are completely powerless.*” (005-F). Also noted by participants was the tension created between potential benefits of involving families with existing privacy and confidentiality considerations, with little room for flexibility. One participant captured this when saying:

*“I feel like the family should be just as involved as the patient. They’re important because they see the symptoms and they live with the individual from day -to-day. And so I think that their very input is just as valid as what the patient is experiencing, but obviously the barrier around protecting PHI and patient rights creates a situation where the family is almost left outside of the care”* (048-SP).

### 3.6 TAY involvement

#### 3.6.1 TAY participating in care

In order for MHA care and transitions to proceed successfully, TAY need to be willing to access care and meaningfully engage in their care. One participant identified this need when saying, “*they need to want to get help or there’s not much you can do*” (023-F). TAY participation in care may be especially difficult if TAY do not recognize the presence of an MHA concern or do not feel able to participate in care. As one caregiver participant described, “*I had a challenge having her agree that she needed care. She was adamant that she wasn’t depressed*” (004-F). Another caregiver shared, “*my daughter is not invested in treatment and getting well*” (060-F). One TAY participant shared how this can feel:

*“When I found myself in a very negative headspace, the last thing I actually want to do is to get help because I am just so focused with feeling overwhelmed by what I am feeling in that moment. But I don’t have energy to actually go through the process of seeking out someone to help me even though I am fully aware that that person can probably provide a lot of support. So I think it’s just that it’s really challenging to get over that because in the moment you are just so focused on what you are struggling with”* (025-TAY).

#### 3.6.2 TAY-friendly care

In order to support youth participation in care, it is paramount that service providers who work specifically with TAY in the MHA system be responsive and appropriately trained to address youth’s particular needs, preferences, and developmental trajectories. Failure to do so may result in unnecessary interruptions and more frequent transitions in care. One caregiver shared,

*“that’s the other terrifying thing, is how do I find someone my kid can actually relate to? Because some of them are so clinical, they don’t have any ability to relate to these kids. So my younger daughter’s been through 5 or 6 therapists and she thinks she’s broken. She says, ‘there’s something wrong with me because none of these people can help’”* (018-F).

Furthermore, the care environment and modes of support were highlighted as opportunities to engage with youth. For example, one participant expressed, “*I think that when you come into a building and-or when you come into a service that doesn’t stigmatize mental health, or doesn’t penalize you for having mental health or addiction, I think that that’s the number one*” (001-SP). This participant continued, stating that when TAY are:

*“too anxious and they can’t get to that appointment, then they get a phone call saying, ‘well if you don’t make your next one, then you’re not going to be part of this program.’ A majority of times, they don’t show up to the next appointment because they already feel like they’re being disciplined”* (001-SP).

#### 3.6.3 TAY independence

Youth independence is essential for autonomy and self-determination, and should be fostered ahead of upcoming transitions. For example, one service provider shared an experience with a TAY, stating, “*I don’t think she has really had the opportunity to make her own choices. It’s always what does her mom say, what does grandma say*” (061-F). Youth independence should be promoted while balancing family involvement and youth needs in terms of their own wellness, to prevent them being caught off-guard when asked to make their own care decisions. For example, one caregiver participant noted, “*I can’t make the decisions for her. It has to be her and they won’t talk to me and so, there’s that parental thing too where you have got to let go.*” (019-F). Another caregiver highlighted the importance of supporting their youth in gaining independence:

*“When we’re gone, he would be living on his own, so he needs to learn. Our role is to train them to be able to support themselves, to be independent. When they have mental challenges or this anxiety hits them, they know where to seek help, they can go on their own, advocate for themselves”* (021-F).

One service provider described supporting TAY in their independence in care, while balancing family involvement at the same time:

*“Sometimes we have family members involved where, there is an enmeshment with the youth, they are very agreeable to their parents being involved because they are actually not used to doing things on their own…Then I am trying to work on fostering the youth’s independence while also allowing them to have a close relationship with family.”* (041-SP)

## 4 Discussion

This study explored the factors that affect transitions in care for TAY with MHA concerns and their families. TAY, caregivers, and service providers in the study spoke of the various and complex *pathways to care,* the need for *appropriate and comprehensive care*, the importance of *continuity of care*, ensuring *informed care*, and finally, creating space for *TAY involvement* and *family involvement*. These findings highlight the complex interplay of barriers and facilitators along the multitude of transitions TAY and families face in MHA care.

Participants’ expressed needs and experiences regarding MHA care transitions identified how, in MHA systems with a breadth of services, TAY and families feel unsupported and fall through gaps in care. Participants in this study described experiences of *interruptions in care*, periods of time during which they had no MHA support, and a lack of *preparation for transitions*, which culminated in a lack of *continuity of care*. Poor transition experiences have been previously linked to a lack of *continuity in care* for TAY with MHA concerns.^19^, ^20^ Furthermore, *continuity of care* has been identified as a marker of quality for transitions across TAY patient populations, not exclusive to MHA.^21^ Understanding factors contributing to unsuccessful transitions is important, as prior work has identified that these experiences can lead to disengagement from care, either by never finding another service, or accessing another service but discontinuing participation if the service is not appropriate or preferred.^22^ Such difficulties also contribute to TAY ‘bouncing’ from service to service, artificially creating unnecessary transitions, as evidenced by the findings in this study. Experiences of *discontinuity* and lack of communication among providers have also been seen at transition boundaries in other areas of healthcare, such as pediatric rehabilitation.^23^ Altogether, the perceived lack of communication, coordination, and collaboration among providers across the system contributed to these experiences of *interruptions in care*, supporting prior findings in this regard.^20^, ^24^, ^25^ Navigation services are one potential way of addressing these needs and supporting TAY and families through transitions in MHA care.^26^ Future work can explore the impact of services and supports that aid in integration of care and system navigation across transitions and support inter-professional communication.

The difficulties experienced by TAY during transitions in care can invoke *family involvement*, and the findings of this study emphasized the importance of family involvement and support for effective transitions. Family support is vital for TAY, but should also be considered in light of individual TAY and family contexts and preferences.^11^, ^27^ Dimitropoulos and colleagues have also emphasized the importance of collaborative *family involvement* that promotes *TAY autonomy*.^27^ Also worth recognizing is the demands of caregiving taken on by family members when trying to find care and support transitions for TAY with MHA concerns, as well as the barriers they encounter in trying to effectively support TAY in their care, findings which were also corroborated by earlier work.^28^, ^29^ These study findings highlight the importance of focusing on engaging TAY in their care and in transition preparation, as well as supporting involved families throughout the transition process, in order to enhance outcomes for TAY.

While participant accounts conveyed the myriad of barriers experienced through transitions in MHA care, these were also often framed as opportunities to facilitate more effective transitions. For example, participants described the difficulties associated with the lack of *transition planning* encountered, which conveys the importance and need for proactive preparation for upcoming transitions and also aligns with existing literature.^11^, ^20^, ^30^ Structured transition approaches and guidelines, such as the Got Transition initiative or the Health Quality Ontario Standard for Transition from Youth to Adult Health Care Services provide guidance for health care professionals, tools for TAY and families, and measurement guides to support improved practice in this domain.^31^, ^32^ The themes identified in this study represent the needs of TAY and families when they experience transitions in care, and therefore present important considerations for service providers and system decision-makers to apply in their approaches.

### 4.1 Limitations

Participants were recruited via MHA service organizations, and therefore had some connection to services. Therefore, perspectives shared in this study may not reflect those who have never engaged with care or those who have entirely disengaged with care. However, the inclusion of multiple participant groups helps ensure that there is some overlap in perspectives, whereby, for example, caregivers might have been connected with services even though their youth may not have been engaged in care. Future work can consider recruiting specifically from outside of the MHA system to ensure viewpoints reflect those who have been the most disenfranchised by MHA care. Secondly, participants all resided in one province of Canada, with the majority from one (albeit large) jurisdiction. These findings may therefore be linked to the specific MHA system in which these individuals are involved. Nevertheless, aspects of these findings may be relatable for TAY, families, and service providers in other MHA systems across Canada and in other countries, especially as they pertain to experiences of transitions in MHA care and the ways in which support is needed. Furthermore, data was not collected regarding race/ethnicity, socioeconomic status, or sexual orientation of participants. While participants did describe experiences of being racialized or marginalized in MHA care, without sociodemographic data and purposive sampling to include marginalized groups, specific analysis to understand these experiences was not possible. Understanding the experiences of various equity-deserving groups in MHA care transitions is an important step for future work. Finally, the data collection period for this study coincided with the onset of the COVID-19 pandemic. The majority of participants completed interviews after the declaration of the pandemic and widespread shutdowns of in-person activities. Thus, some of the difficulties experienced may have been related to these changes rather than ongoing features of the MHA system. COVID-19 pandemic-related impacts to youth MHA service access and transitions have been explored in other work.^10^, ^33^

### 4.2 Conclusion

Exploring TAY, family, and service provider perspectives on MHA care transitions provided important insights regarding difficulties that arise and mitigating factors during an important time in youths’ lives and in their care trajectories. These findings highlight the impact of the multitude of care transitions experienced by TAY with MHA concerns and their families, and the importance of support for transitions through approaches at the system- and individual-care level. Provincial health care systems can purposefully incorporate these study themes into policies pertaining to TAY MHA care, to ensure transitions are proactively planned to ensure continuity of care, that services are appropriate, accessible, and comprehensive, and that TAY and families have the needed resources and guidance to be included and make informed choices about the TAY’s MHA care. Providing support for TAY with MHA concerns and their families in a manner that takes into account the key thematic findings of this study will serve to ensure efficient transitions and continuity of care, ensuring TAY are able to access care that will help them flourish in this important life stage.

## Ethics statement

All study procedures were performed in compliance with relevant laws and institutional guidelines and have been approved by the Sunnybrook Health Sciences Centre Research Ethics Board (#339–2019, October, 2019). Informed consent was obtained for research with human participants.

## Funding statement

Financial support for the conduct of the research was provided by the Canadian Institutes of Health Research (Funding Reference Number: TEG165589). The funder had no role in study design; collection, analysis, and interpretation of data; in writing the report; or in the decision to submit the article for publication.

## Funding

This work was supported by the Canadian Institutes of Health Research – Transitions in Care Evaluation Grant [funding reference number: TEG165589].

## CRediT authorship contribution statement

**Roula Markoulakis:** Conceptualization, Data curation, Formal analysis, Funding acquisition, Investigation, Methodology, Project administration, Resources, Supervision, Validation, Writing – original draft, Writing – review & editing. **Hinaya Cader:** Data curation, Formal analysis, Validation, Writing – original draft, Writing – review & editing. **Karen Wong:** Formal analysis, Validation, Writing – review & editing. **Sugy Kodeeswaran:** Conceptualization, Funding acquisition, Investigation, Methodology, Project administration, Validation, Resources, Writing – review & editing. **Tracey Addison:** Funding acquisition, Investigation, Methodology, Validation, Writing – review & editing. **Cathy Walsh:** Funding acquisition, Investigation, Methodology, Validation, Writing – review & editing. **Jocelyn Charles:** Funding acquisition, Investigation, Methodology, Validation, Writing – review & editing. **Amy Cheung:** Funding acquisition, Investigation, Methodology, Validation, Writing – review & editing. **Deepy Sur:** Funding acquisition, Investigation, Methodology, Validation, Writing – review & editing. **David Willis:** Funding acquisition, Investigation, Methodology, Validation, Writing – review & editing. **Anthony Levitt:** Conceptualization, Funding acquisition, Investigation, Methodology, Project administration, Validation, Resources, Supervision, Writing – review & editing.

## Declaration of Competing Interest

The authors declare that they have no known competing financial interests or personal relationships that could have appeared to influence the work reported in this paper.

## Data Availability

Data will be made available on request.
