# Supplementary material for: Experiences of barriers and facilitators in mental health care transitions: A qualitative exploration of perspectives from transitional-aged youth, family, and service providers (part 1)
Source: Health Care Transit. 2024 Nov 27;3:100087. doi: 10.1016/j.hctj.2024.100087 (PMC11657471; doi:10.1016/j.hctj.2024.100087)
Supplement: Supplementary file 1 — Supplementary material [file mmc1.docx]

*Supplement 1:* Sample Semi-Structured Interview Guide with Possible Probes (Transitional-Aged Youth Interviews)

| Question 1: | **Tell me a little about why you are interested in this study.** |
| --- | --- |
| Probes: | Why is this important to you?  Can you tell me more about that? |
| Question 2: | **What has it been like for you when trying to find mental health and/or addiction services?** |
| Probes: | Can you give me an example? How does it feel?  What has it been like moving from one type of service to another? Finding adult services/the next needed service?  Tell me more about that.  How do you know what type of care you needed? What made you realize? |
| Question 3: | **Do you think there are any challenges when trying to get help?** |
| Probes: | Can you tell me more about that? What challenges have you faced?  Can you give me an example? How does it feel?  Information/communication/costs/knowing where to look/service “fit”/locations/hours/formats |
| Question 4: | **Has there been anything that made it easier for you to get MHA care?** |
| Probes: | In what ways has your family been involved?  How would you like your family to be involved?  Can you tell me more about that?  What has made it easier to move from child to adult services/to the next needed service after one wraps up? To move from one type of service to another?  What has worked and what hasn’t worked?  Can you give me an example? How does it feel? |
| Question 5: | **Was there anyone/anything who helped you connect with a service or move from one service to another?** |
| Probes: | Can you give me an example? How does it feel?  Yes - What was helpful about it?  No - What would’ve been helpful?  How was it once you got there? What was helpful/not helpful?  (Are you feeling okay with the questions so far? Do you wish to continue with the interview?) |
| Question 6: | **Where would you go if you needed to find MHA services in the community?** |
| Probes: | If unsure, how would you go about figuring out where to find services? |
| Question 7 | **Do you know anything about navigation?** |
| Probes: | What do you know about it? Have you had any experience with it?  Is it helpful?  Do you see a role for navigation in the MHA system?  What about it is helpful? Not helpful? |
| Question 8: | **How has it been like trying to find help for physical health issues?** |
| Probes: | Do you see differences in access between MHA and physical health care?  Can you give me an example? How does it feel? |
| Question 9: | **Is there anything not available in the system that would be helpful for you to access MHA care or move from one type of care to another?** |
| Probes: | Can be idealistic  Can you give me an example? |
